# Proteomic Analyses of the Vitreous Humour

**DOI:** 10.1155/2012/148039

**Published:** 2012-08-29

**Authors:** Martina Angi, Helen Kalirai, Sarah E. Coupland, Bertil E. Damato, Francesco Semeraro, Mario R. Romano

**Affiliations:** ^1^Liverpool Ocular Oncology Research Group, Department of Molecular and Clinical Cancer Medicine, Institute of Translational Medicine, University of Liverpool, Liverpool, UK; ^2^Department of Ophthalmology, Spedali Civili di Brescia, University of Brescia, Brescia, Italy; ^3^Department of Ophthalmology, Istituto Clinico Humanitas, Milan, Italy

## Abstract

The human vitreous humour (VH) is a transparent, highly hydrated gel, which occupies the posterior segment of the eye between the lens and the retina. Physiological and pathological conditions of the retina are reflected in the protein composition of the VH, which can be sampled as part of routine surgical procedures. Historically, many studies have investigated levels of individual proteins in VH from healthy and diseased eyes. In the last decade, proteomics analyses have been performed to characterise the proteome of the human VH and explore networks of functionally related proteins, providing insight into the aetiology of diabetic retinopathy and proliferative vitreoretinopathy. Recent proteomic studies on the VH from animal models of autoimmune uveitis have identified new signalling pathways associated to autoimmune triggers and intravitreal inflammation. This paper aims to guide biological scientists through the different proteomic techniques that have been used to analyse the VH and present future perspectives for the study of intravitreal inflammation using proteomic analyses.

## 1. Introduction

The human vitreous humour (VH) is a transparent, highly-hydrated gel, which occupies the posterior segment of the eye between the lens and the retina [1]. It is comprised almost entirely of water (99%) with the remainder consisting of a mixture of collagen fibres, hyaluronic acid, hyalocytes, inorganic salts, and lipids [2]. The average protein concentration of the healthy VH is 0.5 mg/mL, consisting largely of albumin (60–70%). Further components are globulins, coagulation proteins, complement factors, and low-molecular-weight proteins [3]. The ciliary body provides a constant fluid exchange by diffusion, ultrafiltration, and active transport of aqueous fluid into the posterior segment [4]. Proteins may accumulate in the vitreous by local secretion (e.g., glycoprotein), filtration from blood (e.g., albumin), or diffusion from the surrounding tissues [5]. Because of the close contact between the vitreous and the inner retina, physiological and pathological conditions of the retina affect both the proteome and the biochemical properties of the VH. Various vitreoretinal diseases induce changes in specific vitreous proteins, especially when the blood-retinal barrier is disrupted [6].

Because VH can be totally or partially removed without marked detriment to the eye [1], surgical vitrectomy and vitreous biopsies are performed as part of routine clinical practice, providing abundance of human VH samples for analysis. Many earlier studies investigated levels of individual proteins in VH from healthy and diseased eyes, using biochemical or immunological techniques, in particular enzyme-linked immunoabsorbent assay (ELISA) [7–10]. This approach, however, is not suitable for the discovery of networks of functionally related proteins; hence it can further our understanding of the pathophysiology of a disease only to a limited degree.

Proteomics is the large-scale study of the entire complement of proteins, the so-called proteome, present in a cell, tissue, biofluid, or organism in any given state [11]. A novel hypothesis can be generated from global protein expression analysis of disease tissue, which can then be addressed with cellular and in vivo functional studies. Proteomic analyses of healthy and diseased VH have been performed [5, 6, 10, 12–24] to scrutinize the protein profile of vitreoretinal diseases, with the ultimate aim of identifying disease markers that could become the diagnostic and pharmaceutical targets of the future. The search so far has not been conclusive, but as proteomics is still an evolving field, better technologies and deeper understanding of the peculiar nature of the VH bear promising potential.

This paper aims to guide biological scientists through the different proteomic techniques that have been used to analyse the VH. It will discuss their findings and limitations. A second objective is to present future perspectives for the study of intravitreal inflammation using proteomics.

## 2. Proteomic Workflow

Proteomics experiments are categorised according to their objective: assay or discovery. Assay or targeted studies typically seek to quantify a predefined set of proteins or peptides, whereas discovery experiments aim to analyse larger, “unbiased” sets of proteins [11]. All proteomic analyses conducted on VH have used mass spectrometric discovery techniques to facilitate the identification and quantification of the many proteins occurring in the VH, expanding the spectrum of suitable candidates for targeted analyses.

Of the discovery methods that have been developed, all involve a multistep process, which includes sample acquisition, digestion of the protein sample into peptides, fractionation of the peptide mixture (or prefractionation of the proteins, depending on the technique chosen), protein identification by mass spectrometry, and data analysis. The various methods differ in their requirements for sample preparation, the extent and the level of sample fractionation (proteins or peptides), the type of MS, and the data processing tool used [25].

Each step will be described, reporting the different experimental strategies used for analysis of the VH and discussing their advantages and limitations.

## 3. Sample Acquisition

### 3.1. Anatomical Considerations

Anatomically, the vitreous body can be subdivided into three main regions: the vitreous core, the vitreous base, and the vitreous cortex. The vitreous core (or central vitreous) comprises the main bulk of the VH and is a highly hydrated extracellular matrix, which is normally a cellular. The vitreous base and cortex both contain a low concentration of cells, named hyalocytes, and dense bundles of collagen fibrils [1].

Skeie and Mahajan recently demonstrated by one-dimensional (1D) sodium dodecyl sulphate-polyacrylamide gel electrophoresis (SDS-PAGE) that the different substructures of the human vitreous, when individually isolated from post-mortem eyes, are characterised by an unique protein profile [26]. Hence, the dissection technique and the size of the sample are likely to influence the proteome composition.

### 3.2. Vitreous Sample Collection

For ethical reasons, it is not possible to obtain human vitreous samples from healthy eyes. Vitreous surgery necessitates a pathological state, even in retinal conditions such as macular pucker or macular hole. For this reason, some authors [5] argue that examining VH from a carefully selected biobank eye is more representative of the “normal” vitreous proteome. Whilst such opinion is debatable because of the postmortem changes that can occur, being able to harvest the entire vitreous body offers a definite advantage over the small sample produced by a core vitreous biopsy.

VH can also be extracted from eyes enucleated because of a trauma or an ocular malignancy. In such cases, it is important to preserve the integrity of the globe for pathological examination. In the authors' experience, the majority of the VH can be harvested anyway using a 23 G needle on a 10 mL syringe, which is inserted transclerally in the posterior segment of the intact globe. This yields at least 3 mL (out of the 4 mL total volume of the vitreous body). It is not advisable to harvest the VH following sectioning of an enucleated eye, as on opening the globe the more liquid part of the VH tends to spill, leaving the scientist with a highly viscous residue, which is nonrepresentative.

In the vast majority of studies undiluted core vitreous biopsies are taken at the time of surgical vitrectomy for an underlying vitreoretinal disease, most often proliferative diabetic retinopathy. Approximately 1 mL of undiluted VH can be obtained at the onset of pars plana vitrectomy, with closed infusion line, by manual aspiration with cutting on through the vitrectomy probe into a 2.5 mL syringe connected along the aspiration line. Core vitreous biopsies from patients undergoing vitrectomy for macular hole (MH) have been often used as “normal” controls, as MH is an idiopathic condition that develops as the result of vitreofoveal traction and is therefore unlikely to affect the protein composition of the VH [27].

Most proteomic studies have been conducted on vitreous fluid obtained from diabetic patients undergoing surgery for proliferative diabetic retinopathy (PDR), which is a major cause of vitreous haemorrhage. This is an important element to consider when collecting vitreous fluid for proteomic analyses, as the haemorrhage can cause a massive influx of serum proteins into the VH, confounding results. For this reason, Simó and colleagues have measured vitreous haemoglobin levels with a spectrophotometer and excluded all samples containing more than 5 mg/mL of haemoglobin [14, 19].

The preservation of biological state and sample quality prior to proteomic processing and analysis are extremely important. The proteins should be protected against loss or change as a consequence of proteolytic degradation. Ideally, VH should be snap-frozen in liquid nitrogen immediately and stored at −80°C until used [28]. Some authors recommend adding protease inhibitor cocktail to the VH sample prior to freezing [18].

### 3.3. Vitreous Sample Preparation

The ability to extract proteins is the key limiting factor in all subsequent proteomic identification and profoundly influences differential protein identification associated with diseased states [29]. The main problem when handling VH specimens is the viscous nature of such samples.

The collagen fibrillar network and associated surface macromolecules maintain the VH in a gel state. With age, the vitreous undergoes progressive liquefaction, starting in the vitreous core as pockets of fluid that then coalesce [30]. Neal et al. have measured the viscosity coefficient of different regions of the human VH in phakic and pseudophakic donor eyes. In phakic eyes, viscosity is higher near the lens than near the retina, whilst this trend is reversed in pseudophakic ones [17]. Hence, the macromolecular composition and the viscosity of VH samples differ according to the anatomical region where the sample is taken, the age of the patient, the state of the lens, and the presence of any vitreous pathology.

Viscosity prevents accurate pipetting, posing a problem when small accurate aliquots are needed for antibody-based assays or for assessing the protein content of a large specimen (e.g., Bradford assay) prior to proteomic analyses. Various preanalytical treatments have been proposed to reduce viscosity, including boiling, high-speed centrifugation, microfiltration, dilution, and hyaluronidase treatment [31, 32]. The effect of these treatments on the VH has been investigated in forensic science for the postmortem analysis of chemical analytes such as glucose, urea, and creatinine, but there is no comparative study on the effect of such pre-treatments on proteins.

High-speed centrifugation (12000 rpm for 15 minutes) is the most common technique that is used to separate the liquid component of the VH from its structural one [22]. Centrifugal filters, such as the 0.22 *μ*m GV DURAPORE filter (Millipore, Carrigtwohill, Cork, Ireland) have also been used to clarify vitreous samples [15].

## 4. Fractionation

Because proteomes are very complex mixtures, a number of techniques have been employed to extract them prior to analysis.

*Protein* fractionation is an important first step in facilitating access to the low abundant proteins of interest for clinical research. The most common techniques for this purpose are affinity chromatography for protein depletion and gel electrophoresis for protein separation.

*Peptide *fractionation is used in “shotgun proteomics” where the entire proteome is digested into peptides, which are then fractionated and identified by MS. This approach is thought to introduce less bias into a biological sample; hence it is most frequently used in quantitative protein expression profiling. Column chromatography plays a major role in this phase.

### 4.1. Depletion of Highly Abundant Proteins

Albumin and immunoglobulin account for over 80% of the whole-vitreous protein content, possibly preventing the detection of less abundant proteins. This is particularly relevant in 2D-PAGE experiments, when large spots of albumin and immunoglobulin can overlap small spots, thereby obscuring less abundant proteins. Affinity chromatography is frequently used in proteomic studies of body fluids to deplete highly abundant proteins and enhance the detection of low abundance ones. In VH, IgG removal prior to electrophoresis has been achieved using Protein A Sepharose 4 Fast Flow (Amersham Pharmacia Biotech) [21] or with the ProteoExtract Albumin/IgG Removal Kit (Calbiochem, San Diego, CA, USA) [15].

Immunoaffinity subtraction (IS) is an alternative approach that allows bounding and retrieval of the 12 most abundant plasma proteins (HSA, IgG, fibrinogen, transferrin, IgA, IgM, apolipoprotein A-I, apolipoprotein A-II, haptoglobin, *α*1-antitrypsin, *α*1-acid glycoprotein, and *α*2-macroglobulin) from biological fluids using a commercially available system (Beckman Coulter ProteomeLab IgY-12 column, Beckman Coulter, Fullerton, CA, USA). Kim et al. treated VH samples from eyes with PDR using IgY-12 columns and subsequently compared the low and high abundance protein fractions obtained by 2-DE [15]. Forty-seven spots were excised from the low abundance protein gel and 5 proteins were identified, while 116 spots were excised from the high abundance protein gel and 25 proteins were identified. The identification rate was low in the low abundance protein gel, hence the authors abandoned this prefractionation technique suggesting that high abundance proteins account for the most protein in VH and that low abundance proteins of interest may have also been removed by the IS column, as verified in other studies [33].

### 4.2. Protein Separation by Gel Electrophoresis

SDS-PAGE separates proteins according to their electrophoretic mobility. The sample is first denatured with a buffer containing SDS, which charges each protein with a negative charge, identical per unit mass, so that the electrophoretic run leads to fractionation based solely on size. Depending on gel size and resolution, SDS-PAGE enables separation of proteins into about 10–50 fractions, which are recovered by excision and digested into peptides for sequencing by MS.

For separation of complex protein mixtures with a higher resolution, SDS-PAGE has been combined with isoelectric focusing (IEF), which separates proteins based on isoelectric points. This is called two-dimensional (2D) gel electrophoresis and has been used for several decades in proteomics. The use of immobilised pH gradient strips for IEF is an improved technique that allows resolution of hundreds of denatured proteins in a single 2-DE gel [34]. After electrophoresis, the proteins in the gel are stained for visualisation, quantification, and comparison. The various detection methods (radioactivity, dyes, fluorescence, and silver) as well as the data analysis issues that must be taken into account when quantitative comparative analysis of 2D gels is performed have been critically reviewed in a recent work [35].

2-DE has been the prefractionation technique of choice in the majority of proteomic studies on VH conducted to date [5, 6, 15, 17, 18, 21, 23, 24]. The stain and detection software used evolved over time, moving from Coomassie Brilliant Blue (CBB) for global protein detection [23] to fluorescent dyes with higher sensitivity and dynamic range such as SYPRO Ruby protein stain [18]. Relative quantification of protein expression levels between samples was estimated based on the assumption that the optical density of the spots (OD%) had to be proportional to the protein concentration. Differences in apparent protein expression levels between the VH samples were considered potentially significant when matched spots exhibited at least a twofold difference in their averaged OD%. Using this technique, Ouchi et al. performed the first quantitative comparison of 2D gel protein expression in vitreous from patients with and without diabetic macular oedema (DMO), detecting 72 spots from DMO VH and 64 spots from non-DMO VH. The intensity of 8 spot was significantly different, leading to the identification of six proteins (PEDF, apolipoprotein A4, apolipoprotein 1, thyroid hormone receptor interacting protein-11, plasma retinol-binding protein, and vitamin D-binding protein) with higher expression in the DMO group [18].

A more reliable and reproducible method of relative protein quantitation from two or more samples is 2D fluorescence difference gel electrophoresis (DIGE), a version of 2D-PAGE where the proteins of each sample are labelled with a different fluorophore prior to electrophoresis [36]. Gels are scanned at wavelengths unique to each fluorescent label and the images are analysed for differences in protein patterns such as spot density or mass shift.

Using DIGE, Hernández et al. compared VH from eight diabetic patients with DME and eight nondiabetic controls and detected 1300 protein spots. The analysis of spots of differing intensity leads to the identification of 25 proteins, four of which were specifically associated with DMO [37]. García-Ramírez et al. had been the first to apply DIGE for analysis of the VH. Using this technique, they identified 11 proteins as differentially produced in the VH of PDR patients in comparison with VH from non-diabetic subjects; 8 were overproduced (ZAG, apolipoprotein A1, apolipoprotein H, fibrinogen A, C4b, factor B, C3, and C9) and 3 were significantly under produced (PEDF, IRBP, and ITIH2) [14]. The higher expression of apoliprotein A1 and H in PDR patients has been confirmed in a later study by the same group by DIGE and Western blot of VH samples, as well as mRNA expression in the retina [19].

## 5. Protein Identification

Mass spectrometry (MS) is the key analytical technique in proteomics for the identification and, increasingly, for the quantification of proteins. The principle of MS is to measure the mass (*m*) to charge (*z*) ratio of ions in the gas phase, hence the peptides need to be first transferred into the gas phase and ionised.

The two relevant techniques for ionization of peptides, proteins, and protein-like molecules (e.g., glycoproteins) are matrix-assisted laser desorption/ionization (MALDI) [38] and electrospray ionization (ESI) [39]. For MALDI, the analyte is dissolved and cocrystallised with a matrix on a probe surface, which is then irradiated by a UV laser pulses. The laser evaporates and converts analyte into gas phase at the ion source. The ionised analyte is then separated by the time-of-flight (TOF) analyser, most commonly employed in MALDI-MS. The *m*/*z* value of peptides is measured by recording the time ions require to travel over a fixed distance inside the mass analyser. In ESI, the peptide mixture is dissolved in a liquid solvent system instead of the matrix. Highly charged analyte droplets from a fine spray outlet are ionised at atmospheric pressure in the presence of a strong electric field, to generate a series of charged gas-phase ions. The charged ions are then emitted and focused into the high-vacuum region of the mass analyser, which records the various charge states of the molecule separated according to their *m*/*z* ratios. There are a number of mass analysers in addition to the above-described TOF: quadrupole, ion trap, orbitrap, and fourier transform cyclotron ion resonance (FT-ICR). Each one works differently, having their own strengths and weaknesses and can be used alone or in combination [40].

The mass spectra can be directly compared with protein databases for matching the molecular weights using appropriated scoring algorithm (peptide mass fingerprinting) [41]. This technique, however, is limited by the database (as it should contain prior information on the protein for matching) and by the complexity of the protein mixture (as it becomes difficult to select the right peptide mass from a lot of peaks) [42]. Tandem mass spectrometry (MS/MS) involves two consecutive steps: peptide mass determination and generation of partial amino acid sequence information for a particular peptide based on further fragmentation. The *m*/*z* values of the fragments are then recorded in the tandem mass spectrum. Tandem MS can be done by two separate analysers (e.g., TOF-TOF) or inside the same mass analyser (e.g., ion trap).

To enhance detection of proteins from very complex mixtures, frequently used platforms are the LC-MS/MS instruments, where ion-pair reversed chromatography or nanohigh performance liquid chromatography (HPLC) is used prior to tandem MS [43]. Advances in LC-MS/MS have greatly improved the dynamic range and sensitivity for analysis of complex protein mixtures [44]. Large-scale proteome profiling has been verified for different organisms, as well as mammalian tissues and cell lines by using multi-dimensional LC-MS/MS [45]. By adopting this technique, Yu et al. have scrutinised the protein profiles of VH from 24 patients undergoing vitrectomy for proliferative vitreous retinopathy (PVR) and 8 biobank eyes, identifying 363 proteins [22]. An even better example of how proteomics is strictly dependent on the technology employed has been provided by Kim et al., who could identify 49 proteins using 2-DE and 531 proteins using LC-MS/MS on the same set of VH from PDR eyes [15].

## 6. Data Analysis

Algorithms have been developed for amino acid sequence and protein identification by matching the information contained in mass spectra against a database of theoretical or previously identified spectra. Algorithms can generate both false-positive and false-negative assignments, which are influenced by the stringency of spectra to sequence criteria. Discerning a true match from a false match is critical in proteomic data analysis. The most common tools for MS/MS-based peptide identification and data analysis have been comprehensively reviewed elsewhere [46].

Because of the complexity of the proteomic workflow and data analysis, it is essential to validate the identified candidate proteins using independent techniques, such as Western blot. Moreover, the experimental design needs to take into consideration the influence of technical and biological variabilities, which are particularly relevant in biological samples like the VH.

## 7. Previous Studies of the Vitreous Proteome

Fifteen studies conducted over the last decade have used a range of proteomic methodologies including 2DE, DIGE, ESI-MS, MALDI-MS, and LC-MS/MS to compare the vitreous proteome of patients with various stages of diabetic retinopathy (DR) and PVR to that from non-diabetic patients and those with MH [5, 6, 10, 12–16, 18–24, 37]. One other study investigated the proteome of VH from human phakic and pseudophakic donor eyes [17]. In general, the total protein content reported for the vitreous of patients with DR is higher than that measured in the non-diabetic and control samples. As already discussed above, this may be due, however, to an influx of serum due to vitreous haemorrhage and/or disruption of the blood-retinal barrier, leading to elevated levels of proteins not associated with intravitreal protein production. Indeed, in the study of Simó et al., a comparison of proliferative vitreoretinopathy and normal vitreous demonstrated upregulated levels of intraocularly produced lipoproteins in the former [19]. Overall, studies analysing the vitreous proteome in patients with DR have varied greatly both in terms of the total number of proteins identified and the number of proteins differentially expressed between the test group(s) and controls, as well as the specific proteins then proposed to play a role in the pathogenesis of vitreoretinal disease states. Although a detailed discussion of the specific proteins identified by these studies is beyond the scope of this chapter, it is clear that as proteomic technologies have evolved over this period, so the number of identified proteins has increased. Whether any of these proteins and the pathways that they regulate is of importance in the pathogenesis of DR remains a very interesting translational question, which is being investigated by more quantitative targeted approaches.

## 8. Future Perspectives: Proteomics for Intravitreal Inflammation

Intraocular inflammation accounts for 10–15% of bilateral and 22% of unilateral blindness in the United States, and 10% of visual impaired registration in the UK [47]. Many efforts are being made to deepen our understanding of the different aspects of the inflammatory process, evaluate new therapeutic strategies, and ultimately be able to deliver personalised care for patients with intraocular inflammatory diseases [48]. Animal models play a fundamental role in this process [49]. Proteomics analyses of intravitreal inflammation have not yet been performed on human samples, whilst they have been successfully performed on VH from animal models.

Endotoxin-induced uveitis (EIU) is an animal model of acute ocular inflammation. To characterize the mechanism of EIU, Bahk et al. analysed the infiltration of proteins in the vitreous bodies of rats with EIU and normal rats using 2-DE-MALDI-TOF/MS and micro LC/LC-MS/MS, identifying specific modifications in the crystallin family proteins [50].

Spontaneous equine recurrent uveitis (ERU) is a recurrent uveitis that develops in the horse and results in blindness [51]. It is the only spontaneous disease model for human autoimmune uveitis. The vitreous is the body fluid closest to the disease-affected tissue and possibly also an effector of pathological processes relevant for ERU. Surgical removal of the VH can lead to a considerable decrease in the frequency and severity of relapses, therefore vitreous composites are likely to contribute to disease progression [52]. Deeg and coworkers have been systematically comparing VH from healthy and disease-affected equine eyes by proteomic profiling [53, 54]. In an earlier study, they applied 2-DE-MALDI-TOF/MS, identifying a total of 42 proteins, 9 of which differentially expressed in ERU. These are functionally related to immune response, inflammation, and maintenance of the blood-retinal barrier [52]. More recently, they identified ERU-related functional protein networks and affiliated molecular signalling pathways using LC-MS/MS-based label-free quantification followed by pathway enrichment analyses [54]. The increased sensitivity gained by omitting gel-based prefractionation resulted in overall detection of 119 different proteins. A large fraction of these proteins were differentially expressed in ERU samples as opposed to controls (26 upregulated, 44 downregulated). Pathway enrichment analyses were performed using the ConsensusPathDB program, suggesting the participation of the Wnt pathway in the pathogenesis of the uveitis.

This shows how the development of MS-based methods significantly improved quantitative proteomic analyses of the VH, enabling comprehensive identification of differentially regulated proteins and detection of novel molecular pathways that could become the therapeutic targets of the future.
